# Supplementary material for: Causal interplay between lactose intolerance and gut microbiota: a combined bidirectional Mendelian randomization and in vivo validation study
Source: Front Nutr. 2026 Jun 1;13:1803337. doi: 10.3389/fnut.2026.1803337 (PMC13265576; doi:10.3389/fnut.2026.1803337)
Supplement: Supplementary file 1 [file Data_Sheet_1.zip › supplementary materials/Forward/leave one out cross validation/ebi-a-GCST90027482.finngen_R12_E4_LACTONAS.pdf]

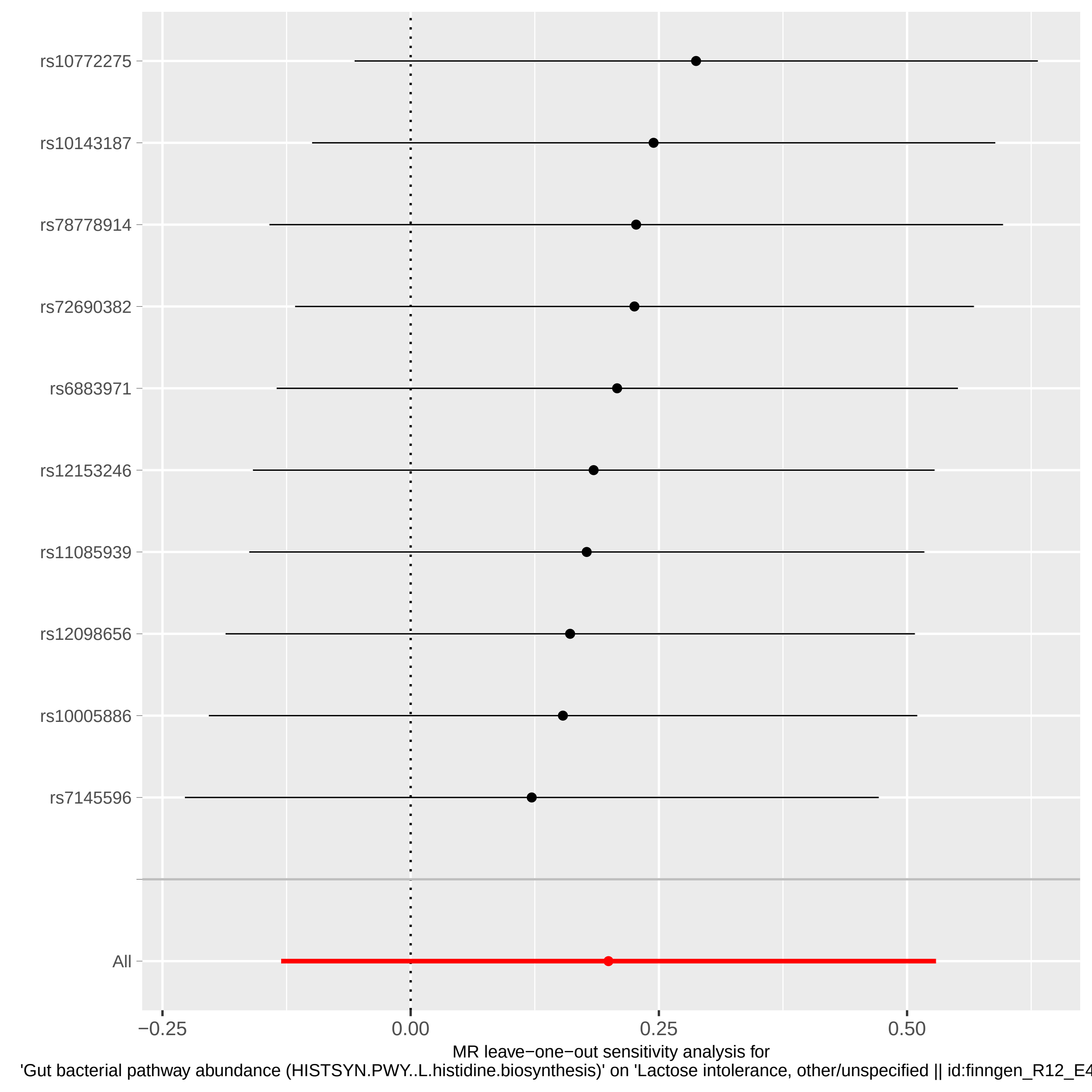
rs10772275
rs10143187
rs78778914
rs72690382
rs6883971
rs12153246
rs11085939
rs12098656
rs10005886
rs7145596
All
−0.25
0.00
0.25
0.50
MR leave−one−out sensitivity analysis for
'Gut bacterial pathway abundance (HISTSYN.PWY..L.histidine.biosynthesis)' on 'Lactose intolerance, other/unspecified || id:finngen_R12_E4
